# Spliced XBP1 Levels Determine Sensitivity of Multiple Myeloma Cells to Proteasome Inhibitor Bortezomib Independent of the Unfolded Protein Response Mediator GRP78

**DOI:** 10.3389/fonc.2019.01530

**Published:** 2020-01-22

**Authors:** Bojana Borjan, Johann Kern, Normann Steiner, Eberhard Gunsilius, Dominik Wolf, Gerold Untergasser

**Affiliations:** ^1^Department of Internal Medicine V, Innsbruck Medical University, Innsbruck, Austria; ^2^Experimental Oncogenomics Group, Tyrolean Cancer Research Institute, Innsbruck, Austria

**Keywords:** myeloma, bortezomib, UPR, p53, resistance

## Abstract

**Background:** Mechanisms mediating resistance against the proteasome inhibition by bortezomib (BTZ) in multiple myeloma (MM) cells are still unclear. We analyzed the activation of the unfolded protein response (UPR), induction of prosurvival, and apoptotic pathways after proteasome inhibition in BTZ-sensitive and -resistant cells. Thereafter, these findings from tissue culture were proofed on MM cells of BTZ-sensitive and BTZ-refractory patients.

**Methods:** Proteasomal and ABC transporter activities were measured in sensitive and resistant cell lines by the use of the respective substrates. TP53 gene loss and mutations were determined by cytogenetics and targeted NGS. UPR pathways, proteasome subunit levels and protein secretion were studied by Western Blot analysis, and apoptosis was determined by flow cytometry. MM cell lines were stably transfected with inducible GRP78 expression to study unfolded protein expression. Transient knock-down of GRP78 was done by RNA interference. Splicing of XBP1 and expression of GRP78 was studied by real-time PCR in CD138-enriched MM primary cells of BTZ-refractory and -sensitive patients.

**Results:** BTZ-sensitive cells displayed lower basal proteasomal activities. Similar activities of all three major ABC transporter proteins were detected in BTZ-sensitive and resistant cells. Sensitive cells showed deficiencies in triggering canonical prosurvival UPR provoked by endoplasmic reticulum (ER) stress induction. BTZ treatment did not increase unfolded protein levels or induced GRP78-mediated UPR. BTZ-resistant cells and BTZ-refractory patients exhibited lower sXBP1 levels. Apoptosis of BTZ-sensitive cells was correlating with induction of p53 and NOXA. Tumor cytogenetics and NGS analysis revealed more frequent *TP53* deletions and mutations in BTZ-refractory MM patients.

**Conclusions:** We identified low sXBP1 levels and *TP53* abnormalities as factors correlating with bortezomib resistance in MM. Therefore, determination of sXBP1 levels and *TP53* status prior to BTZ treatment in MM may be beneficial to predict BTZ resistance.

## Background

Multiple myeloma (MM) is a malignancy of terminally differentiated immunoglobulin-secreting plasma cells and resides predominantly in the bone marrow. Although the introduction of the reversible proteasome inhibitor bortezomib (BTZ) has resulted in the improved survival of MM patients, drug resistance has emerged as an insurmountable obstacle (1, 2). Furthermore, BTZ did not demonstrate success in treating solid tumors (3). The underlying mechanisms of resistance in MM and a variety of solid tumors are still a matter of debate.

Malignant plasma cells produce large amounts of immunoglobulins and thus are considered to be heavily reliant on the protein processing pathways, including the secretion and endoplasmic reticulum-associated degradation (ERAD) pathways (4, 5). Following endoplasmic reticulum (ER) quality control, proteins are destined for either a secretory pathway or degradation by the ubiquitin-proteasome system (UPS) (6). Bortezomib, a boronic acid dipeptide, reversibly inhibits the β5 subunit of proteasome and its chymotrypsin-like (CT-L) activity, thereby inducing the accumulation of proteins (7). Mutations and overexpression of the PSMB5 (β5) gene were observed *in vitro* in BTZ-adapted myeloma cell lines (8), but never *in vivo* in MM patients refractory to BTZ (9).

Large amounts of misfolded proteins induce stress in the ER and activate the unfolded protein response (UPR) that restores protein homeostasis and contributes to cell survival (10). The main signaling regulator of UPR, the chaperone GRP78 (78 kDa glucose-regulated protein), also known as BiP (immunoglobulin binding protein), senses misfolded proteins and assists in their folding and transport to ERAD (11). The persistent disturbance of the protein folding activates terminal UPR and subsequently causes cell death (12). Several hypotheses have been proposed to explain the anti-myeloma activity of BTZ, including the induction of terminal UPR (13), inhibition of NFκB (14), stabilization of pro-apoptotic p53 (15), induction of NOXA (16), and inhibition of multiple cellular proteases (17).

Despite considerable attention being paid to elucidating mechanisms mediating BTZ resistance, the complex underlying processes responsible for intrinsic and acquired resistance in cancer patients are still not well understood (3). Therefore, we investigated the link between proteasome, secretome, unfolded proteins, UPR molecules, and p53/NOXA mediated apoptosis in primary and acquired BTZ resistance. Based on our *in vitro* findings, we analyzed CD138-sorted MM cells from patients with acquired resistance in order to understand the impact of sXBP1, GRP78, and p53/NOXA in therapy responses after proteasome inhibition.

## Methods

### Patient Samples

Patients with newly diagnosed MM (NDMM) and relapsed/refractory MM (RRMM) according to the International Myeloma Working Group (IMWG) criteria were included in the study population (Table S1). Investigations have been approved by the committee of Ethics of the Medical University Innsbruck (AN2015-0034 346/4.13; AN5064 Innsbruck) after obtaining written informed consent for usage of routine samples for the scientific project. All NDMM patients showed response to bortezomib therapy when evaluated 6 months after treatment initiation. Multiple myeloma cells were purified *ex vivo* from isolated bone marrow mononuclear cells using CD138 microbeads (Miltenyi Biotec), and peripheral blood B-cells were sorted using CD19 microbeads (Miltenyi Biotec). The presence of deletion 17p was assessed by interphase fluorescent *in situ* hybridization (FISH) in all MM samples.

### Cell Culture

The BTZ-sensitive multiple myeloma cell lines (OPM-2, NCI-H929, U266, and MM1.S), BTZ-resistant adenocarcinomas of the breast (MDA-MB-231), colon (HRT-18), and prostate (PC-3), and primary foreskin fibroblasts (PFF) used in the study were all authenticated by STR profiling.

### DNA Extraction and Next-Generation Sequencing

Mutational status of TP53 gene was further analyzed by next-generation sequencing (NGS). Genomic DNA was extracted from CD138 enriched cells and tumor cell lines. Thirty nanograms of genomic DNA were used to generate libraries for NGS analysis. Paired-end sequencing was performed with the Miseq Reagent Kit V2 on the Miseq NGS machine (Illumina). NGS results of TP53 mutational status can be found in Table S2.

### Proteasome Activity Assay

To determine the ß5 subunit proteasome activity, a reagent containing luminescent substrate specific for the chymotrypsin-like site, Suc-LLVY-Glo™, was added to living cells with an intact membrane structure or cell extracts after cell lysis, and luminescence was recorded by an Infinite 200 luminometer (Tecan).

### Drug Efflux Assay

Functional profiling of the activity of three major ABC transporters (p-glycoprotein, MRP1/2 and BCRP) was performed using an eFluxx-ID Green multidrug resistance assay kit (Enzo Life Sciences, USA), according to the manufacturer's instructions.

### Generation of Tetracycline-Inducible Lentiviral GRP78-FLAG Overexpression System in Myeloma Cells

Myeloma cell lines (OPM-2^TetR^ and U266^TetR^) were generated as described (18). OPM-2^TetR^ and U266^TetR^ lines were further lentivirally transfected (pLenti6.3/GRP78-FLAG), and stable cell lines were selected over several weeks with 2.5 μg/ml blasticidin (Invitrogen).

### Immunofluorescence and Confocal Microscopy

Myeloma cell line (OPM-2^TetRGRP78−FLAG^) was exposed to 10 μg/ml tetracycline and allowed to express GRP78-FLAG for 48 h. Myeloma cell suspension was stained with mouse anti-FLAG antibody (F1804, Sigma; AB_262044). Imaging was conducted using a spinning disc confocal microscopic system with Velocity software (Ultra VIEW VoX; Perkin Elmer).

### RNA Interference

Two independent siRNA sequences, siGRP78#7 and siGRP78#9, targeting different regions of the GRP78 mRNA, were used to knockdown gene expression in MDA-MB-231, HRT-18 and PC-3 cells.

### Immunoprecipitation of Unfolded Proteins

To detect unfolded proteins in ER, stable myeloma cell line OPM-2^TetRGRP78−FLAG^ with tetracycline-inducible overexpression was stimulated for 48 h to express GRP78-FLAG prior to addition of BTZ for the time course. Cells were harvested using diluted low detergent RIPA Buffer (0.01% NP-40, Cell Signaling) and then subjected to immunoprecipitation using anti-FLAG M2 affinity gel (A2220, Sigma-Aldrich). Thereafter, the and the misfolded immunoglobulin λ light chains were analyzed.

### Statistical Analysis

Statistical analyses were performed with the GraphPad Prism™ (SCR_002798) software for Windows. Student's *T*-test 2-tailed, two-way ANOVA, and Mann–Whitney *U*-Tests were used to study differences between groups.

A more detailed description of all methods used in the project are available in Supplementary Materials.

## Results

### BTZ-Sensitive MM Cells Are More Sensitive to Disturbance of the ER Ca^2+^

**Figure 1:**
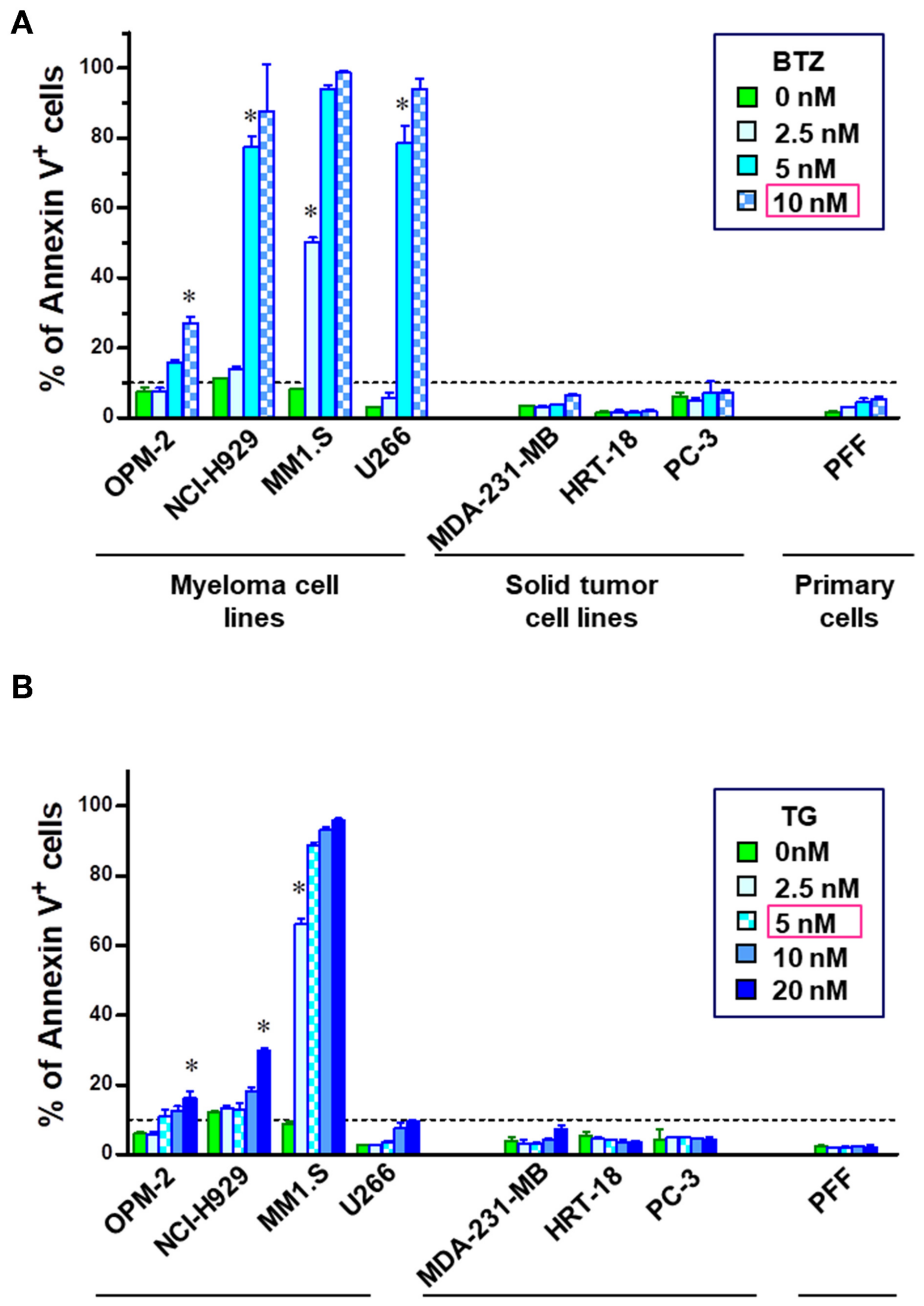
MM cells are sensitive to inhibition of proteasome and disturbance of endoplasmic calcium homeostasis. To examine the apoptosis-inducing potential of bortezomib (BTZ) and thapsigargin (TG), cells were treated with increasing doses of the drug for 24 h. Flow cytometric analysis of cell death in multiple myeloma cell lines (OPM-2, NCI-H929, MM1.S, and U266), solid tumor cell lines (MDA-MB-231, HRT-18, PC-3) and primary fibroblasts (PFF). Apoptosis was determined using Annexin V-PerCP eFluor 710 staining of externalized phosphatidylserine. The cells were incubated with increasing concentrations of the **(A)** BTZ, reversible proteasome inhibitor or **(B)** thapsigargin, a drug disturbing Ca^2±^ homeostasis of the endoplasmic reticulum. *p < 0.05, i.e., first concentration with significant cell death. Statistical analyses were performed with the GraphPad Prism™ software for Windows. Student's *T*-test 2-tailed, two-way ANOVA, and Mann–Whitney *U*-Tests were used to study differences between groups.

Although promising in preclinical experiments, bortezomib failed in almost all of the clinical trials in non-hematological cancers and refractory MM patients develop resistance after prolonged treatment. Therefore, we reanalyzed apoptosis induction potency of BTZ in different cancer cell lines *in vitro*. Four multiple myeloma cell lines (OPM-2, NCI-H929, MM1.S, and U266), three solid tumor cell lines (MDA-MB-231, HRT-18 and PC-3), and human primary fibroblasts (PFF) were chosen to determine the sensitivity to proteasome inhibition by BTZ and ER stress induced by thapsigargin. Myeloma cells exhibited significantly higher sensitivity to proteasome inhibition (Figure 1A) and ER Ca^2+^ disturbance (Figure 1B) than both solid tumors and normal cells. Interestingly, U266 MM cells were resistant to apoptosis when exposed to ER stress. Based on these results, the first lethal concentrations of BTZ (10 nM) and TG (5 nM) was used in further experiments on MM cells avoiding of target effects caused by high drug concentrations.

A fast proliferation rate and turnover of cell cycle proteins have been previously accounted as most likely responsible for enhanced sensitivity to proteasome inhibition. By directly comparing the doubling time of MM and solid tumor cell lines, we found longer cell population doublings in BTZ-sensitive MM cells (Figure S1). Thus, high cell proliferation, i.e., higher turnover of proteasome-dependent cell-cycle proteins, does not make tumor cells more susceptible to proteasome inhibition. Proteasome level and capacity were further analyzed.

### BTZ-Sensitive MM Cells Have Completely Blocked Chymotrypsin-Like Proteasome Activity

**Figure 2:**
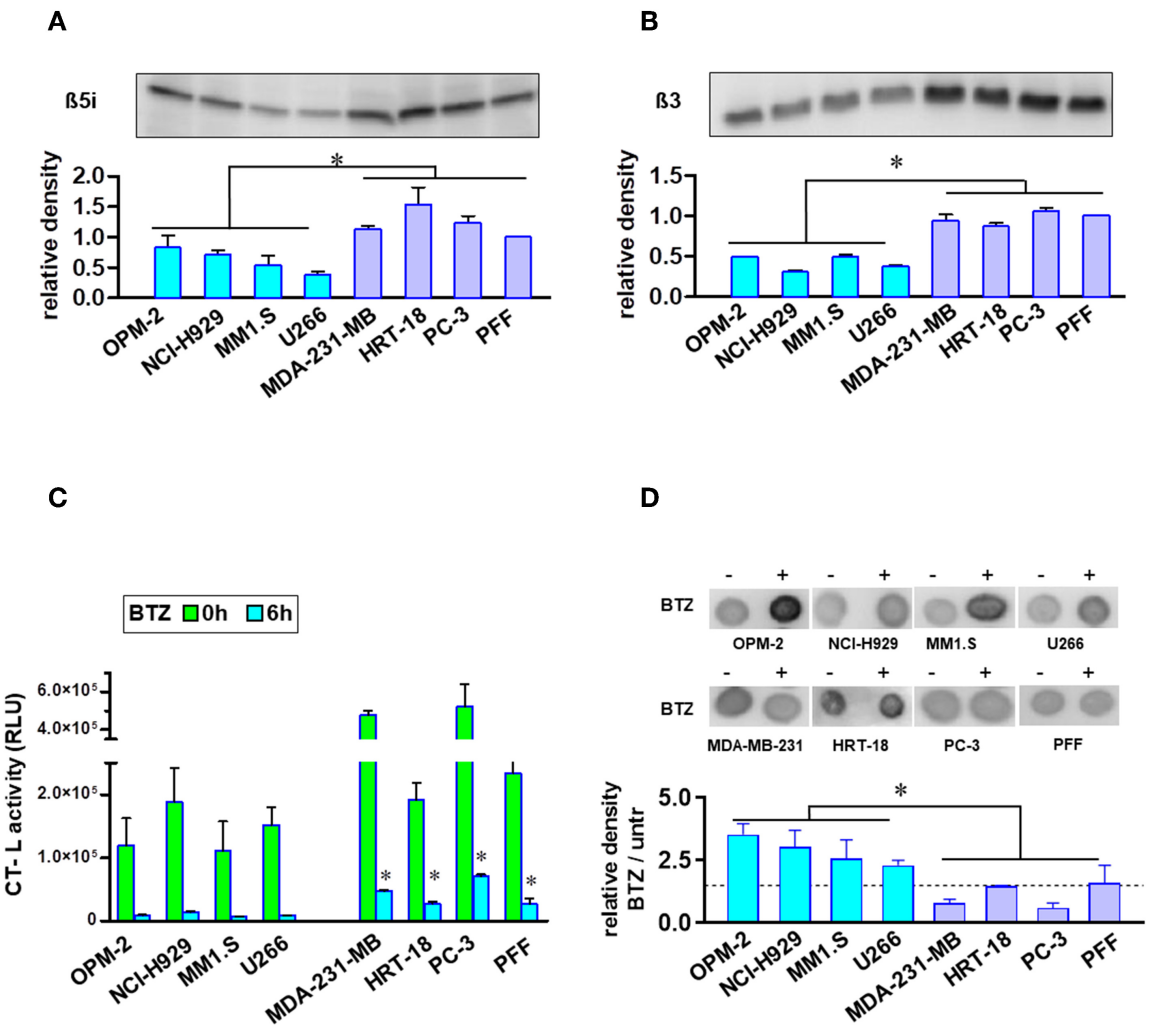
Analysis of proteasome levels, activity, and ubiquitinated proteins in MM and solid tumor cell lines. Proteasome core subunit expression beta 5i **(A)** and beta 3 **(B)** was analyzed by Western Blot (10^5^ cells per lane). Experiments were repeated three times to calculate relative expression normalized to cell number and compared with reference control PFF (mean expression was set to 1). In comparison to BTZ-resistant solid tumor cell lines and fibroblasts, MM cells displayed lower proteasomal subunit expression when correlated to equal cell numbers. **(C)** Equal cell numbers of MM and solid tumor cell lines were exposed to BTZ (10 nM) for 6 h. Thereafter, chymotrypsin-like (CT-L) activity was detected by an assay with viable cells monitoring cleavage of substrate peptide after uptake. CT-L activity is displayed as relative luminescence unit (RLU). BTZ-resistant cells display significant higher residual CT-L activity after BTZ-treatment. **(D)** Dot blot analysis of the accumulation of ubiquitinated proteins in MM and solid tumor cells after treatment with the proteasome inhibitor. Equal cell numbers of MM and solid tumor cell lines were exposed for 6 h to BTZ (10 nM). Thereafter, proteins were analyzed by an anti-Ubiquitin Lys-48 specific antibody in a Dot Blot analysis and subsequently quantified. Mean of three experiments ± SD is shown. *p < 0.05.

Proteasome amount and activity were compared using equal numbers of tumor or normal cells. MM cell lines showed significantly lower protein expression of β5i and β3 proteasome subunits (Figures 2A,B). Additionally, MM cells had no significant increased levels of ubiquitinated proteins (Figure S2). Finally, we measured the uptake of BTZ and subsequent inhibition of basal CT-L activities in living cells with an intact membrane structure (Figure 2C). Regarding basal CT-L activity, the addition of BTZ for 6 h resulted in completely suppressed CT-L activity in MM cells, whereas solid tumors and fibroblasts still maintained significantly higher residual CT-L activity (Figure 2C). The inhibition of the proteasome β5 subunit significantly elevated the ubiquitinated protein levels in MM cells, whereas solid tumor cell lines and primary fibroblasts displayed no significant accumulation (Figure 2D). Interestingly, respective extracts after cell lysis and subsequent exposure to BTZ showed the same rate of inhibition of CT-L activity in all cell lines (Figure S3), implying differences in uptake or efflux between MM and solid tumor cells. However, drug efflux by three major ABC transporters (p-glycoprotein, MRP1/2 and BCRP) did not notably differ between BTZ sensitive and resistant cell lines (Figure S4).

### BTZ-Sensitive MM Cells Do Not Have Higher Protein Synthesis and Secretions

Myeloma cells, which synthesize large amounts of unfolded and misfolded immunoglobulins, are considered to be heavily dependent on ER, UPR, and UPS. This vulnerability of malignant plasma cells is exploited as a treatment target and used to partially explain the success of BTZ in MM therapy. To assess the rate of protein synthesis and secretion, we compared the amounts of secreted proteins in MM and solid tumor cells lines after normalization to equal cell numbers. Analysis of the conditioned medium and the total protein extracts suggests that resistant solid tumors synthesize and secrete amounts of proteins that do not differ significantly from those of MM cells (Figure S5).

### BTZ Treatment Does Not Lead to Elevated Unfolded Immunoglobulins Levels in the ER

**Figure 3:**
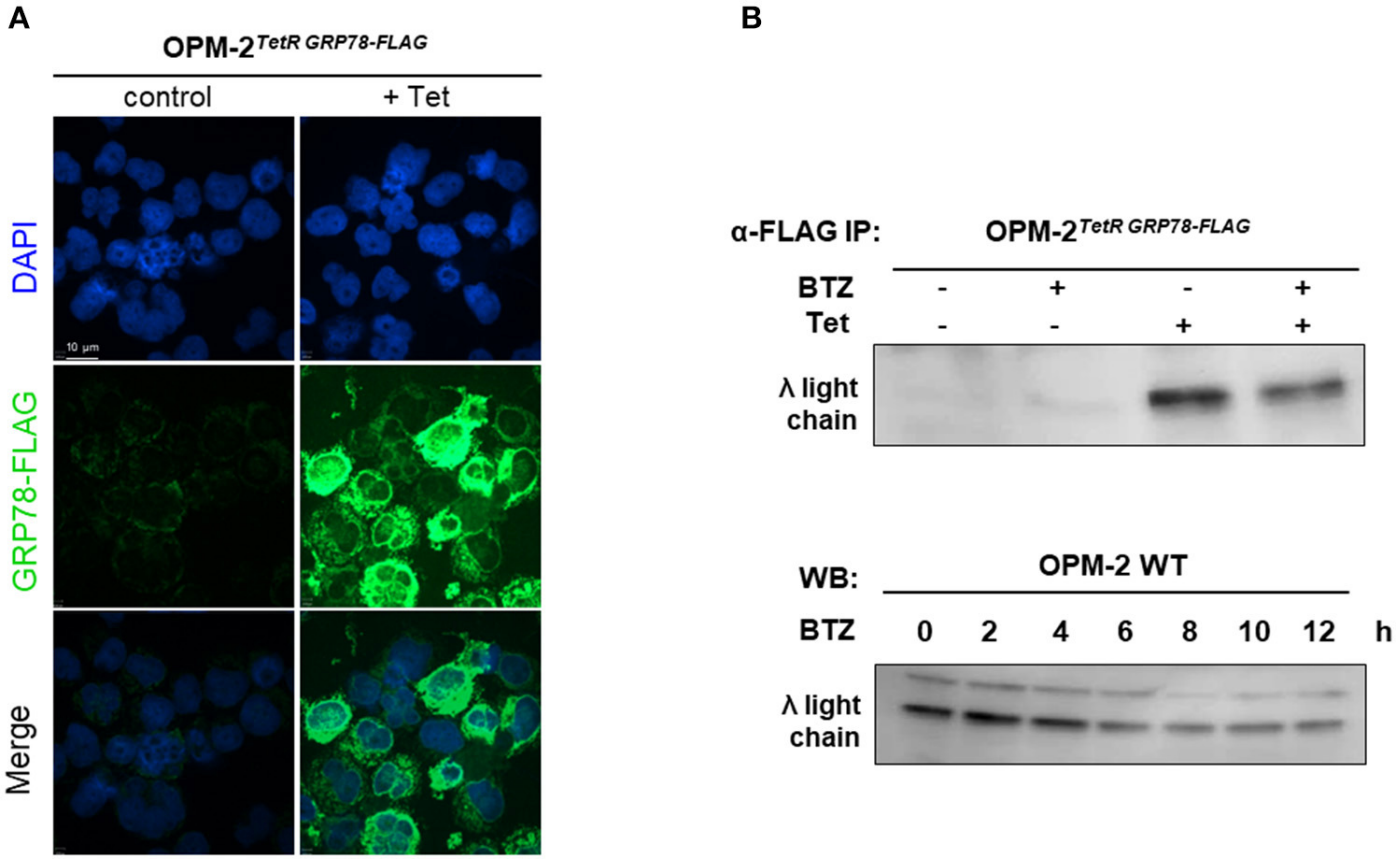
Proteasome inhibition does not lead to higher amounts of unfolded proteins in MM cell line. **(A)** OPM-2^TetRGRP78−FLAG^ were stimulated with 10 μg/mL tetracycline for 48 h to achieve a robust expression of GRP78-FLAG. Confocal immunofluorescence shows inducible GRP78-FLAG expression stained by anti-FLAG primary antibody. The nuclei are counterstained with DAPI (blue). Scale bar represents 10 μm. **(B)** After 48 h of tetracycline induction, OPM-2^TetRGRP78−FLAG^ were treated with 10 nM BTZ for additional 6 h. Cell lysates were immunoprecipitated (IP) with anti-FLAG antibody to enrich co-precipitated unfolded proteins like the λ light chains, which subsequently were detected by Western Blot analysis. **(C)** Western Blot analysis of OMP-2^WT^ cells displaying total intracellular λ light chain protein levels in a time course up to 12 h after treatment with 10 nM BTZ. A representative result of three independent experiments is shown.

Since we found no correlation between protein secretion rates and sensitivity to BTZ, we hypothesized that BTZ treatment of MM cells may lead to a higher concentration of unfolded proteins intracellularly. Therefore, we explored the accumulation of misfolded proteins, particularly λ light immunoglobulin chains in the ER of MM cell lines. Due to the lack of commercially available detection systems and methods, we created an immunoprecipitation (IP)-based system for monitoring unfolded proteins. Our system is based on the ability of chaperone GRP78 to bind misfolded proteins during their synthesis and UPR in the ER. We established a tetracycline-inducible lentiviral system for the overexpression of GRP78-FLAG in OPM-2 MM cells. A robust induction of GRP78-FLAG in OPM-2^TetR GRP78−FLAG^ clones 48 h after tetracycline treatment, has been shown by immunofluorescent staining (Figure 3A). Treatment with BTZ did not result in significantly higher levels of misfolded λ light chains in the ER of the myeloma cells as detected by α-FLAG IP before the occurrence of apoptosis (Figure 3B). Moreover, we also could not detect a significant increase in λ light chains by using a Western blot on the total cell extracts in a time course up to 12 h after BTZ stimulation (Figure 3C). Furthermore, overexpression of GRP78, a major UPR molecule, did not rescue myeloma cells from BTZ-induced apoptosis (Figure S6), but knockdown of GRP78 in BTZ-resistant solid tumor cells induced apoptosis (Figure S7).

### BTZ-Sensitive MM Cells Have Deficiencies in Responding by UPR After Disturbance of the ER Ca^2+^ Homeostasis

**Figure 4:**
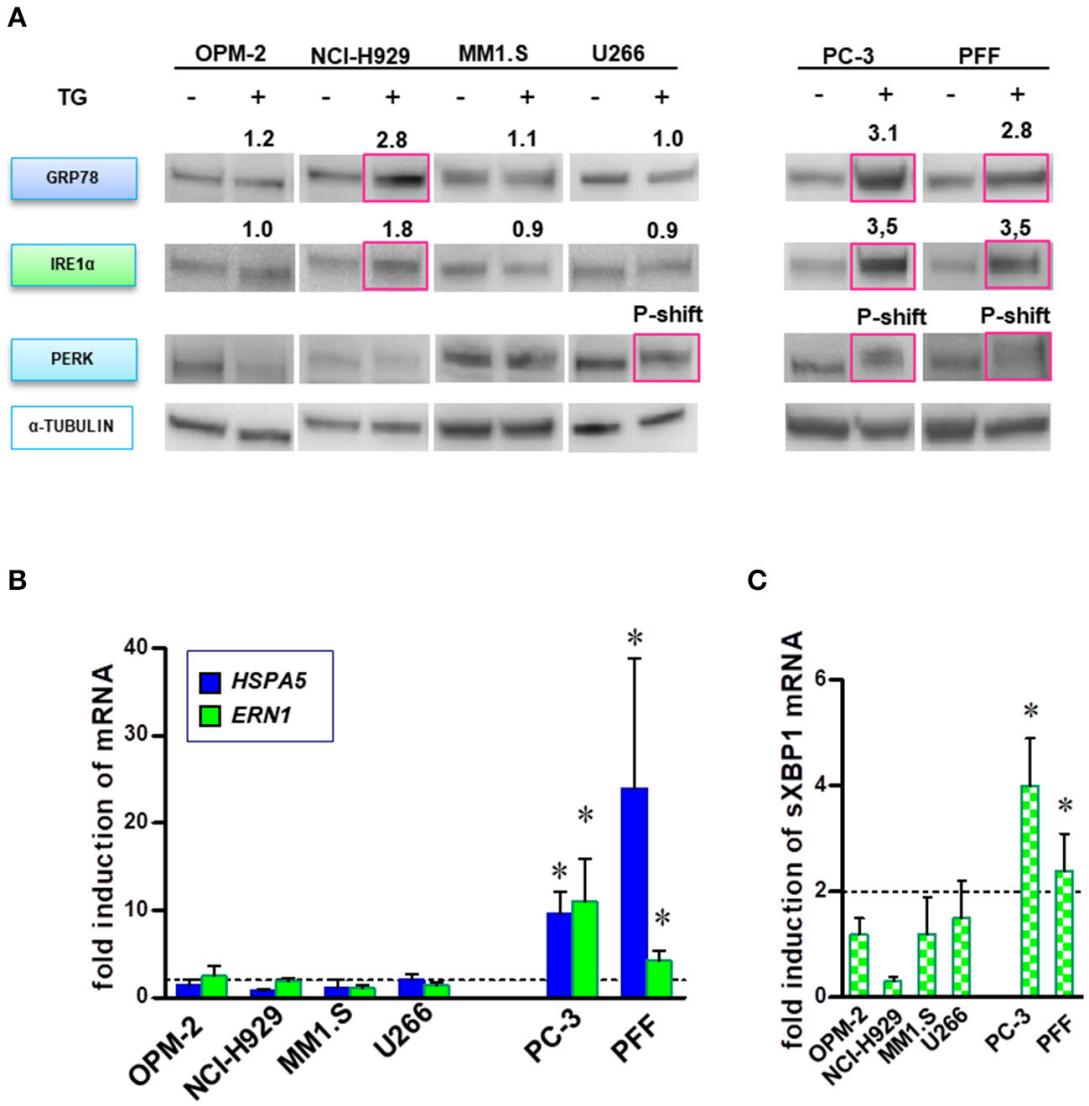
MM cells lack full ability to respond by canonical UPR prosurvival signaling after disturbance of ER homeostasis. MM, PC-3 and PFF cells were treated with TG (5 nM), and activation of UPR was analyzed in a time course of 3–24 h. **(A)** Proteins specific to the three main branches of UPR (i.e., upregulation of GRP78 and IRE1α, and PERK phosphorylation) were detected by Western blot. The data represents at least three repeated experiments. The red square indicates significant upregulation of protein or phosphorylation-induced mobility shift (p-shift; PERK). Numbers indicate mean upregulation as determined by densitometry of chemoluminescence signals of two experiments. **(B)** Moreover, real-time PCR analysis was performed to monitor changes in *HSPA5* (GRP78) and *ERN*1 (IRE1α) gene expression after BTZ-treatment. Mean ± SD of three independent experiments. **(C)** Splicing of the XBP1 primary transcript was analyzed in MM and solid tumor cell lines after induction of ER stress. Mean ± SD of three independent experiments. *p < 0.05.

Both the activation and disruption of the UPR branches under BTZ treatment have been previously reported (13). We initially explored the functionality of the UPR machinery in our cell lines. First, we induced general ER stress in four MM lines (OPM-2, NCI-H929, MM1.S, and U266), prostate cancer cells (PC-3) and normal fibroblasts by applying thapsigargin, an inhibitor of the endoplasmic Ca^2+^ ATPase (SERCA). Activation of all major transducers of UPR, i.e., upregulation of IRE1α (*ERN1*), GRP78 protein levels and phosphorylation of PERK (Figure 4A), HSPA5 (GRP78) and ERN1 gene expression (Figure 4B) and splicing of XBP1 mRNA (Figure 4C). PC-3 and PFF cells displayed pro-survival signaling with the activation of all three branches of UPR. OPM-2 and MM1.S myeloma cell lines did not activate UPR. Upregulation of GRP78 and IRE1α in NCI-H929 cells did not protect cells from ER stress, mainly due to the absence of the downstream splicing of XBP1. Noticeably, U266 the only MM cell line surviving higher thapsigargin concentrations (Figure 1B), showed phosphorylation of PERK. Furthermore, we determine the capability of proteasome inhibition to induce ER stress and UPR. None of the cell lines, either MM or solid tumors, responded with significant activation/elevation of UPR proteins or mRNA (Figure S8). In summary, most MM cells lack operative pro-survival responses to ER stress.

### High Levels of IRE1α and Spliced XBP1 Correlate With Sensitivity to BTZ-Induced Apoptosis

**Figure 5:**
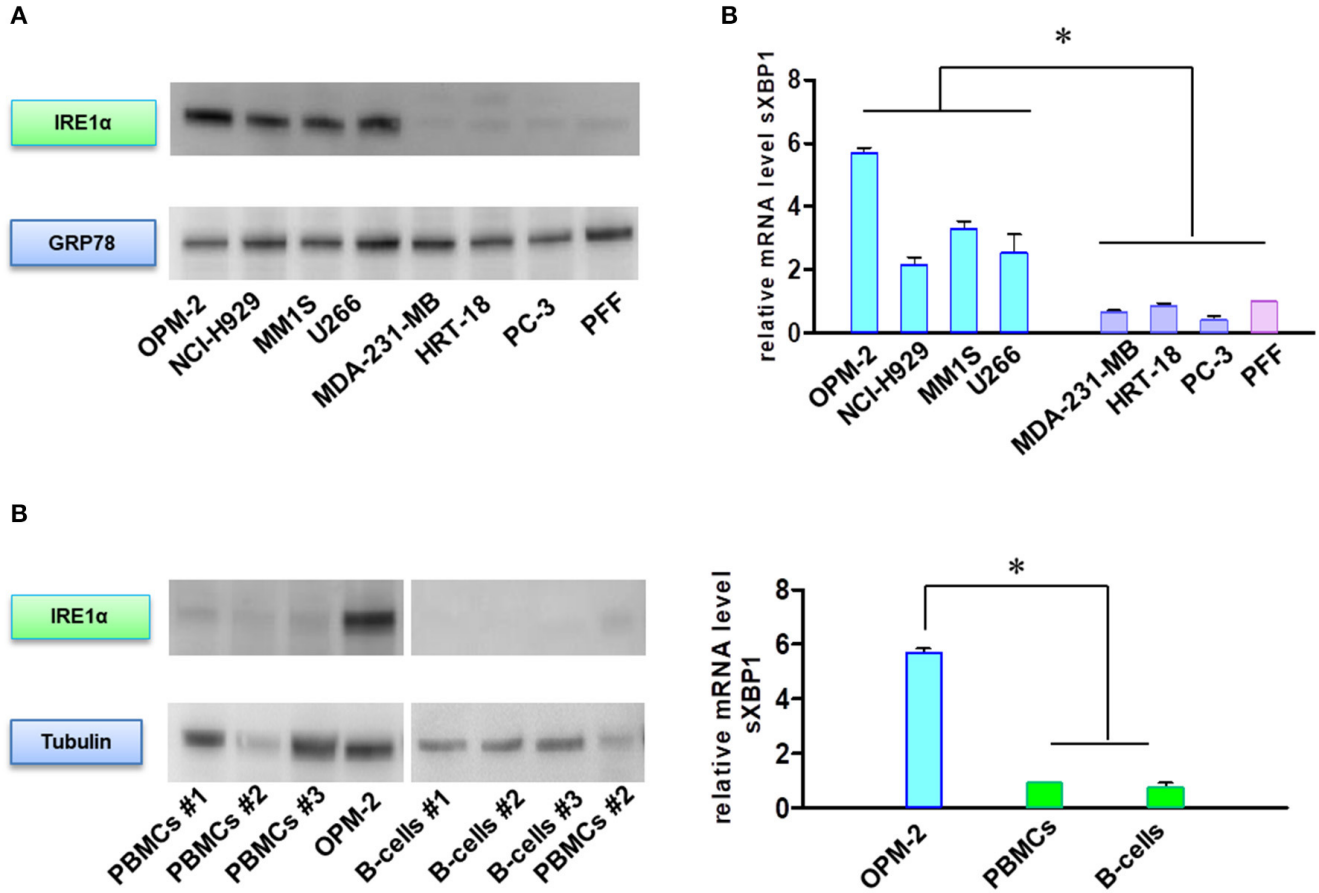
MM cells sensitive to BTZ display high IRE1α and sXBP1 levels. **(A)** Western Blot analysis of IRE1α and GRP78 protein expression in sensitive and resistant cells. **(B)** Western Blot analysis of IRE1α and tubulin protein expression in MM plasma B cells and peripheral blood mononuclear cells (PBMCs) and CD-19 enriched B-cells. **(C)** Spliced XBP1 transcript levels were analyzed in sensitive and resistant cells by real-time PCR. RNA levels in cell lines were normalized to 18S and expression values of PFF (upper panel). RNA levels in cell lines were normalized to 18S and expression values of PBMCs (lower panel). Mean ± SD of three independent experiments. *p < 0.05.

**Figure 6:**
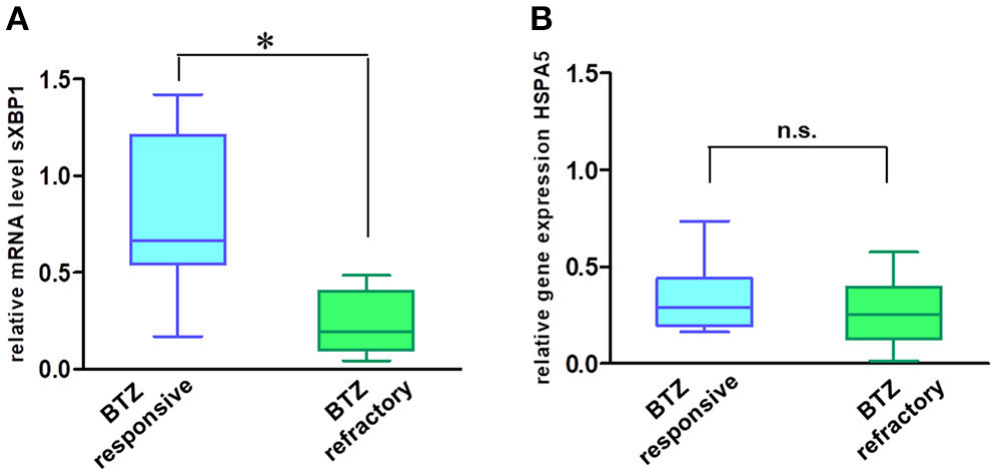
MM cells of patients sensitive to BTZ treatment have high sXBP1 levels. Relative mRNA levels of sXBP1 **(A)** and *HSPA5* gene expression **(B)** were analyzed in CD138^±^ enriched cells isolated from bone marrow aspirates of BTZ-responsive (*n* = 10) and resistant (*n* = 10) MM patients by real-time PCR. RNA levels in cell lines were normalized to ACTB, *p < 0.05.

In addition to their role in transmitting UPR signals, IRE1α and its downstream target XBP1 have an essential role in the plasma cells differentiation from their progenitors (19, 20). Moreover, sXBP1 has an important role in immunoglobulin synthesis and secretion. The protein expression of IRE1α (Figures 5A,B) and mRNA level of sXBP1 (Figure 5C) were uniformly increased in MM cell lines as compared to solid tumors, fibroblasts, peripheral blood mononuclear cells (PBMNCs), and sorted B-cells. This observation seems to be independent of ER stress, since all cell types express similar levels of GRP78, the central sensor of UPR (Figure 5A). Based on this finding, we further freshly isolated CD138 positive bone marrow cells from BTZ-responsive (*n* = 10) and BTZ-refractory (*n* = 10) patients and compared mRNA levels in cancerous plasma cells. Resistance to BTZ treatment was associated with lower mRNA levels of sXBP1 (Figure 6A). Nonetheless, gene expression of *HSPA5* (encoding GRP78) did not differ significantly between BTZ–responsive and BTZ-refractory patients (Figure 6B).

### p53/NOXA Responses Correlate With Sensitivity to BTZ Treatment in MM Patients

**Figure 7:**
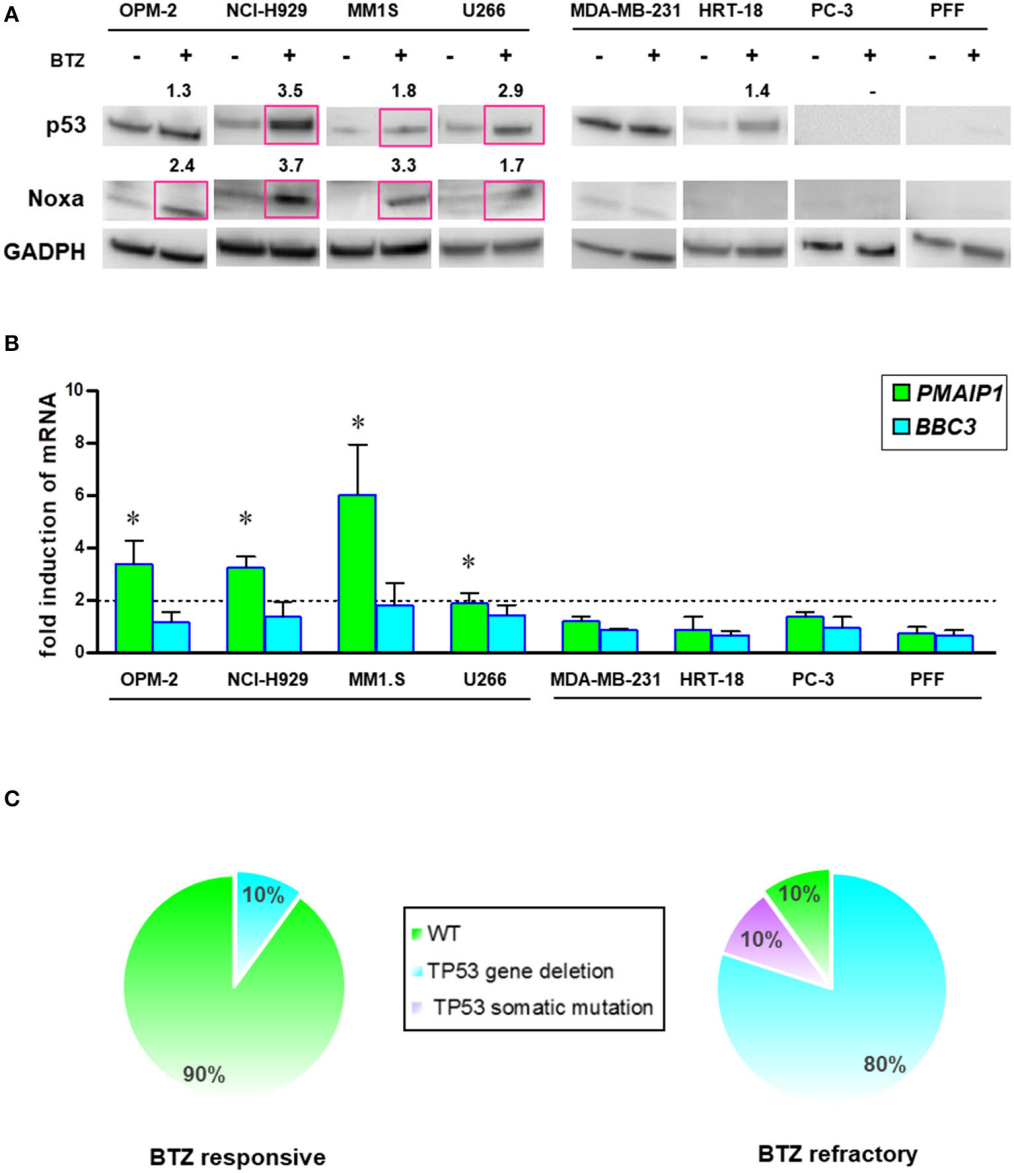
BTZ-sensitive MM cell lines have intact p53/NOXA responses for induction of apoptosis. **(A)** Western Blot analysis of p53 and NOXA protein expression after treatment with BTZ (10 nM, 8 h). *GAPDH* was used as a loading control. The data represents at least three independent experiments. Red squares indicate significant upregulation and mean value after densitometric quantification of chemoluminescence. **(B)** Real time PCR analysis of *PMAIP1* (NOXA) and *BBC3* (Puma) gene expression after treatment with BTZ (10 nM, 8 h). The data is expressed as mean ± SD of three independent experiments. *p < 0.05. **(C)** Frequency of *TP53* mutation and deletions across CD138^±^ cells isolated from bone marrow aspirates from BTZ-responsive (*n* = 10) and refractory (*n* = 10) MM patients. NGS was performed on all patients showing no 17p deletion in cytogenetics. One BTZ-refractory patient (MM#18) with no 17p deletion displayed a M246V TP53 mutation. All NGS results of TP53 mutational status can be found in Table S2.

Intracellular levels of the tumor suppressor p53 are regulated by rapid degradation via proteasome. Loss of *TP53* at the chromosomal level has been implicated in the progression of MM. Therefore, we analyzed the potential correlation of p53/NOXA induction and its response on target gene transcription after BTZ treatment. After treatment with BTZ, MM cell lines increased levels of p53 and NOXA at the protein level (Figure 7A). NOXA mRNA levels were increased in BTZ-sensitive MM cells, but not in solid tumor cells or primary fibroblasts. Puma (*BBC3*) gene expression was not significantly affected in any cell line tested (Figure 7B).

Furthermore, we analyzed the mutation status of the *TP53* gene in all cell lines by NGS (Table S2). We found a positive correlation between mutated *TP53* and the lack of NOXA activation. Induction of *PMAIP1* (NOXA) gene expression in MM cells was associated with wild-type (NCI-H929, MM1.S) or gain-of-function p53 (OPM-2; R175H). The partially functional p53 (T161A) in the U266 myeloma cell line correlated with a lower potency of NOXA protein and mRNA elevation (Figures 7A,B).

Based on this finding, we performed an interphase FISH for the loss of chromosome 17p, and subsequent NGS analysis of the *TP53* gene in our collections of MM cells isolated from the marrow cells of patients responding or refractory to BTZ treatment (Figure 7C). The majority of the patients responding to BTZ treatment exhibited no chromosomal loss or intragenic mutations of the *TP53* gene.

## Discussion

Despite recent developments, the molecular mechanisms leading to resistance to proteasome inhibition by BTZ in multiple myeloma are still controversial (21). In this study, we investigated the underlying mechanisms that could be implicated in mediating the sensitivity or resistance to proteasome inhibition in MM and solid tumors. Previous studies have found mutations in the proteasome subunit PSMB5 (β5) in long-term BTZ-adapted myeloma cell lines, but not in patients refractory to BTZ (8, 22). Therefore, to avoid this weakness of *in vitro* induction of acquired BTZ resistance in myeloma cell lines, we directly compared a panel of sensitive myeloma with intrinsically resistant solid tumor cell lines. We observed differences in BTZ sensitivity. Our data provide some explanations for the failure of BTZ in the treatment of solid tumors, as well as acquired resistance in MM patients.

Consistent with previous findings in PC-3 cells (23), BTZ was able to inhibit proteasome activity in biochemical assays of total cell extracts in all the cell lines tested. Nonetheless, when analyzed in living cells in the presence of the intact membrane structure, we noted the residual activity in BTZ-resistant cells that was sufficient to prevent the accumulation of ubiquitinated proteins. These observations point to either differential uptake or the efflux of BTZ in solid tumor cells. Unfortunately, we were not able to measure the intracellular concentrations of BTZ to prove differences in specific and/or unspecific uptake mechanisms. A study of cellular BTZ kinetics in myeloma cell lines showed no significant impacts by efflux transporters on intracellular drug concentrations (24). However, studies on the contribution of aberrant drug transporters to BTZ resistance are not in complete agreement (25). Our investigations did not reveal a higher activity of multi-drug resistance (MDR) efflux proteins in resistant cell lines. Interestingly, metabolic adaptation and the upregulation of drug transporters in carfilzomib-adapted, but not in BTZ-resistant MM cell lines, has been shown in a recent publication of Soriano et al. (26).

Since plasma cells have the functions of secreting large amounts of immunoglobulins, it has been speculated that these features might be responsible for the high sensitivity to proteasome inhibition and the induction of cell death (27). In fact, a decrease in immunoglobulin synthesis was reported in a resistant BTZ-adapted myeloma cell line (28). According to our data, like MM cells, solid tumor cells contain and secrete a similar or even greater amount of proteins.

Since we did not find alterations in the secretions, we further speculated that the differences in BTZ sensitivity might be caused by the accumulation of misfolded proteins in the expanded ER of plasma cells after a blockade of UPS (29). It has already been reported that BTZ accumulates misfolded λ light chains in the cytosol of myeloma cells and consequently provokes ER stress (13). Due to the lack of available methods appropriate to measure misfolded proteins, we developed a system that detects misfolded proteins in the ER of the myeloma cell line OPM2. Misfolded λ light chains did not accumulate in the ER of our myeloma cells upon proteasome inhibition by BTZ. This is consistent with the data of Anderson et al. who did not observe accumulation of ERAD substrate in the ER of HEK 293FT cells after BTZ treatment (30). Moreover, total λ light chain levels in cell extracts after the inhibition of ERAD at the proteasome did also not significantly increase in our cells.

Our study suggests that the sensitivity of MM cells to ER stress originates from deficiencies in their response by pro-survival signaling. Nonetheless, they have high levels of IRE1α and sXBP1, known factors of UPR that support proliferation and chemoresistance in breast cancer (31, 32). The IRE1α/XBP1 signaling pathway has emerged as an important pathway involved in the pathogenesis of MM (33, 34). High IRE1α/XBP1 levels are a hallmark of plasma myeloma cells and a potential Achilles heel for therapeutic interference (33, 35, 36). Our findings in patients have demonstrated that sensitivity to BTZ correlates with high sXBP1 levels, and therefore is in line with published data (28, 34, 37). Studies showed discrepancies in association of XBP1 mRNA expression levels and response to BTZ-based therapy (28, 35). In comparison with previously used *in situ* hybridization, we established validated RT–PCR for more specific and sensitive detection of spliced XBP1 mRNA (34). Nevertheless, we assume that high levels of IRE1α/sXBP1 in MM cells are rather a marker of plasma cell differentiation than a sign of high ER stress (19, 20, 37). MM patients sensitive or refractory to BTZ have similar levels of the UPR sensor molecule GRP78. Therefore, we conclude that MM cells have no higher basal ER stress than solid tumors or primary cells, but they do have deficiencies in the activation of the pro-survival signaling program of UPR. The high levels of IRE1α/sXBP1 are presumably fully sequestered to maintain the B-cell immunoglobulin production/secretion program, and thus are not available for adequate UPR pro-survival responses. This could explain the high sensitivity of MM cells to ER-stress inducing drugs.

Regarding ER-stress, we did not find the induction of GRP78 protein, PERK phosphorylation or increases of misfolded proteins in MM cells after proteasome inhibition. Even the overexpression of GRP78 as a key regulator of UPR did not protect MM cells from BTZ-induced apoptosis. Previous studies have suggested the important role of GRP78 in cancer cell survival, which agrees with the lethal consequences of the GRP78 knockdown observed in our BTZ resistant solid tumor cell lines. Despite the sensitivity of MM cells to general ER-stress, i.e., thapsigargin, we did not find a classical prolonged UPR in these cell lines after BTZ treatment, as previously reported (13). An explanation for the divergent results might be the clonal heterogeneity of MM, and the generation of sub-clones with and without intact UPR response (38). Moreover, as suggested by the most recent studies, we screened precisely for UPR activation, avoiding the non-specific factors of UPR that can also be activated independently of ER stress, such as peIF2α, CHOP, or ATF4 (39). Further, the absence of the upregulation of GRP78 mRNA demonstrated inactivity of any of the three UPR branches. Initial work in this field suggests impairment of the IRE1α function in BTZ-treated myeloma cell lines (40). In addition, our findings show no activation of any of the three main UPR branches. Nevertheless, the combination of ER-stress inducers and proteasome inhibition is a promising treatment option to eradicate myeloma cells in the bone marrow (20).

Previous studies have shown p53 upregulation upon BTZ treatment in myeloma cell lines, but these studies did not focus on correlations with NOXA and PUMA as its executors (41). In our investigations of MM cells, we found a strong correlation of BTZ resistance and inactivation of the p53/NOXA pathway of apoptosis. However, currently we have no clear evidence that NOXA is the solely executor of BTZ induced apoptosis. Hemizygous deletion of 17p is associated with a partially or severely inactivated p53 response (42). Therefore, we performed NGS analyses of TP53 gene only in patients showing no 17p deletion. Our findings are in agreement with the BTZ induced apoptosis in endothelial cells (43) and indicate the importance of *TP53* mutations and subsequent resistance to pharmacological inhibition of the proteasome (44). Recent NGS analysis and chromosome 17p tumor cytogenetics has revealed that the frequency of *TP53* deletions/mutations steadily increases up to 80% in later MM stages upon prolonged therapy (38, 45). Therefore, determination of sXBP1 levels and *TP53* status prior to BTZ treatment and studies about targeting p53 abnormalities in MM might be beneficial to predict and overcome BTZ resistance.

## Conclusions

The response to BTZ is based on a complete inhibition of the proteasome in MM cells and correlates with intact induction of p53/NOXA mediated apoptosis. In addition, we assume that sensitivity to BTZ treatment depends on the “mature” differentiation status of MM cells reflected by the levels of IRE1α and the transcription factor sXBP1 and that GRP78-mediated UPR has no impact on response to BTZ.

## Data Availability Statement

All datasets generated for this study are included in the article/Supplementary Material.

## Ethics Statement

The studies involving human participants were reviewed and approved by Investigations have been approved by the ethic committee of the Medical University Innsbruck (AN2015-0034 346/4.13; AN5064 Innsbruck). Written informed consent was obtained from all patients for anonymous usage of their diagnostic routine sample for the scientific project. The patients/participants provided their written informed consent to participate in this study.

## Author Contributions

GU designed the study. GU, JK, and BB developed the methodology. BB performed the research. GU, BB, JK, and NS analyzed the data. BB, GU, EG, and DW drafted the manuscript. All authors read and approved the final manuscript.

### Conflict of Interest

The authors declare that the research was conducted in the absence of any commercial or financial relationships that could be construed as a potential conflict of interest.

## Abbreviations

ABC transporter: ATP-binding cassette transporters
BCRP: Breast Cancer Resistance Protein
BiP: Binding immunoglobulin Protein
BTZ: bortezomib
CT-L activity: chymotrypsin-like proteasome activity
GRP78: Glucose-regulated Protein 78
ER: Endoplasmic Reticulum
ERAD: ER-associated degradation
IRE1α: inositol-requiring enzyme 1 α
NF-κB: Nuclear Factor-κB
MM: Multiple Myeloma
MDR: Multi Drug Resistance
MRP1/2: Multidrug resistance-associated protein 1/2
PERK: Protein Kinase R (PKR)-like endoplasmic reticulum kinase
PgP: P-glycoprotein
PFF: Primary Foreskin Fibroblasts
TP53: Tumor Protein p53
UPR: Unfolded Protein Response
UPS: Ubiquitin/Proteasome System
sXBP1: spliced- X-Box binding Protein 1.
