# Supplementary material for: Spliced XBP1 Levels Determine Sensitivity of Multiple Myeloma Cells to Proteasome Inhibitor Bortezomib Independent of the Unfolded Protein Response Mediator GRP78
Source: Front Oncol. 2020 Jan 22;9:1530. doi: 10.3389/fonc.2019.01530 (PMC6987373; doi:10.3389/fonc.2019.01530)
Supplement: Supplementary file 1 [file Table_1.DOCX]

**Table S-1**

| **Parameter** |  | **NDMM** | |  | **RRMM** | |
| --- | --- | --- | --- | --- | --- | --- |
|  |  | n= 10 | % |  | n= 10 | % |
| Median age (range), years |  | 77(47-85) | 100 |  | 63 (50-72) | 100 |
| Sex f/m |  |  |  |  |  |  |
| f |  | 5 | 50 |  | 6 | 60 |
| m |  | 5 | 50 |  | 4 | 40 |
| ISS |  |  |  |  |  |  |
| I |  | 2 | 20 |  | 1 | 10 |
| II |  | 3 | 30 |  | 4 | 40 |
| III |  | 5 | 50 |  | 5 | 50 |
| Cytogenetic standard risk |  | 6 | 60 |  | 2 | 20 |
| Cytogenetic high risk |  | 4 | 40 |  | 8 | 80 |
| Therapy lines |  |  |  |  |  |  |
| 1^st^ line |  | 10 | 100 |  | 0 | 0 |
| 2^nd^ line + 3^rd^ line |  | 0 | 0 |  | 7 | 70 |
| 4^th^ line + subsequent lines |  | 0 | 0 |  | 3 | 30 |
| BTZ responsive |  | 10 | 100 |  | 0 | 0 |
| BTZ refractory |  | 0 | 0 |  | 10 | 100 |
|  |  |  |  |  |  |  |
|  |  |  |  |  |  |  |
|  |  |  |  |  |  |  |
|  |  |  |  |  |  |  |
|  |  |  |  |  |  |  |
|  |  |  |  |  |  |  |
| n, number of patients; ISS, International staging system; BTZ, bortezomib | | | | | | |
|  |  |  |  |  |  |  |

**Patient demographics and characteristics**

**Table S-2**

***TP53* mutational status as determined by targeted NGS Analysis**

| **Cell line** | | **Type** | **Genotype** | **Protein Position** | **Amino Acids** | **Codons** | | **Activity** |
| --- | --- | --- | --- | --- | --- | --- | --- | --- |
| OPM-2 | | snv | hom | 175 | R/H | cGc/cAc | gain of function | |
| NCI-H929 | | snv | hom | 72 | P/R | cCc/cGc | functional | |
| MM1.S | | snv | hom | 72 | P/R | cCc/cGc | functional | |
| U266 | | snv | hom | 161 | A/T | Gcc/Acc | partially-functional | |
|  | |  |  |  |  |  |  | |
| MDA-MB-231 | | snv | hom | 280 | R/K | aGa/aAa | non-functional | |
| HRT-18 | | snv | het | 117 | G/V | gGg/gTg | non-functional | |
| PC-3 | | deletion | hom | 138 | frameshift |  | no protein | |
|  | |  |  |  |  |  |  | |
| PFF | | snv | hom | 72 | P/R | cCc/cGc | functional | |
| MM#18 | | snv | het | 246 | M/V | Atg/Gtg | non-functional | |
|  | snv, single nucleotide variant; hom,homozygote; het, heterozygote | | | | | | | |
|  |  | | | | | | | |
