# Supplementary material for: Spliced XBP1 Levels Determine Sensitivity of Multiple Myeloma Cells to Proteasome Inhibitor Bortezomib Independent of the Unfolded Protein Response Mediator GRP78
Source: Front Oncol. 2020 Jan 22;9:1530. doi: 10.3389/fonc.2019.01530 (PMC6987373; doi:10.3389/fonc.2019.01530)
Supplement: Supplementary file 2 [file Presentation_1.pdf]

Figure S1

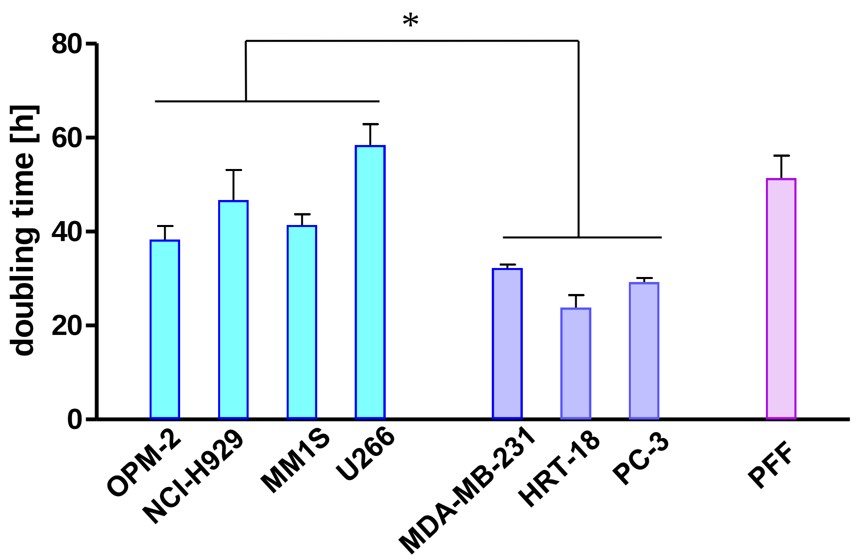

Analysis of cell cycle progression (doubling time) of myeloma and solid tumor cell lines and primary fibroblast.

- indicates $p<0.05$. Statistical analyses were performed with the GraphPad Prism™ software for Windows. Student’s T test 2-tailed, two-way ANOVA and Mann-Whitney U Tests were used to study differences between groups.

MM cell lines have longer cell population doubling times than solid tumor cell lines. Higher mitotic activity is not correlating with sensitivity to proteasome inhibition.

Figure S2

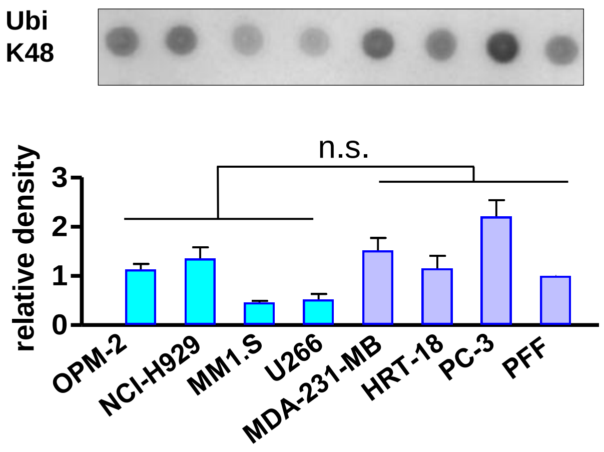

Analysis of ubiquitinated proteins in multiple myeloma and solid tumor cell lines.

Lysin-48-ubiquitin conjugates were studied by Dot Blot analysis after normalization of all cell lines to $5x10^3$ cells per dot. The intensity of each dot was determined by densitometry. Experiments were repeated three times to calculate relative expression normalized to cell number and compared with reference control PFF, which mean expression was set to 1. Quantification analysis is represented by the graph.

MM cell lines have no higher basal levels of ubiquitinated proteins which must be degraded over proteasome.

Figure S3

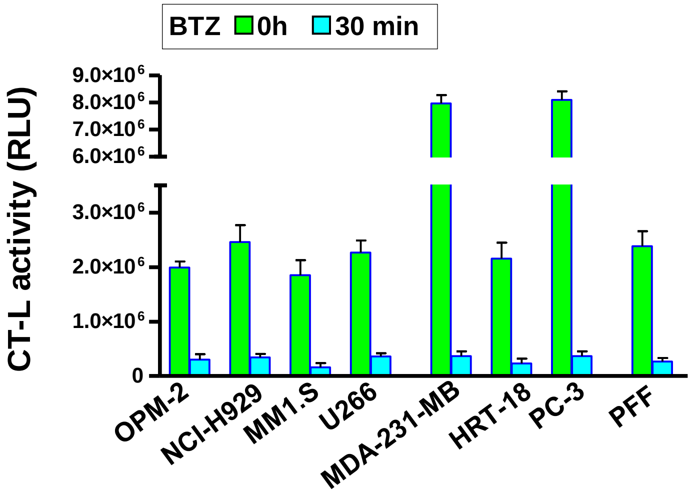

Analysis of CT-L proteasome activity in cytosol extract of MM and solid tumor cell lines

The cytosol extracts of equal cell number of MM, solid tumor cell lines, and fibroblasts were exposed for 30 min to bortezomib (10nM). Thereafter, chymotrypsin-like (CT-L) activity was detected by a biochemical assay for monitoring cleavage of substrate peptide. All myeloma cells, solid tumors and fibroblast showed similar inhibition rates of CT-L activity. CT-L activity is displayed as a relative luminescence unit (RLU). Mean of three replicates ± SD is shown.

BTZ (10 nM) inhibited chymotrypsin-like (CT-L) activity totally in all cell extracts after removal of the cell membrane.

Figure S4

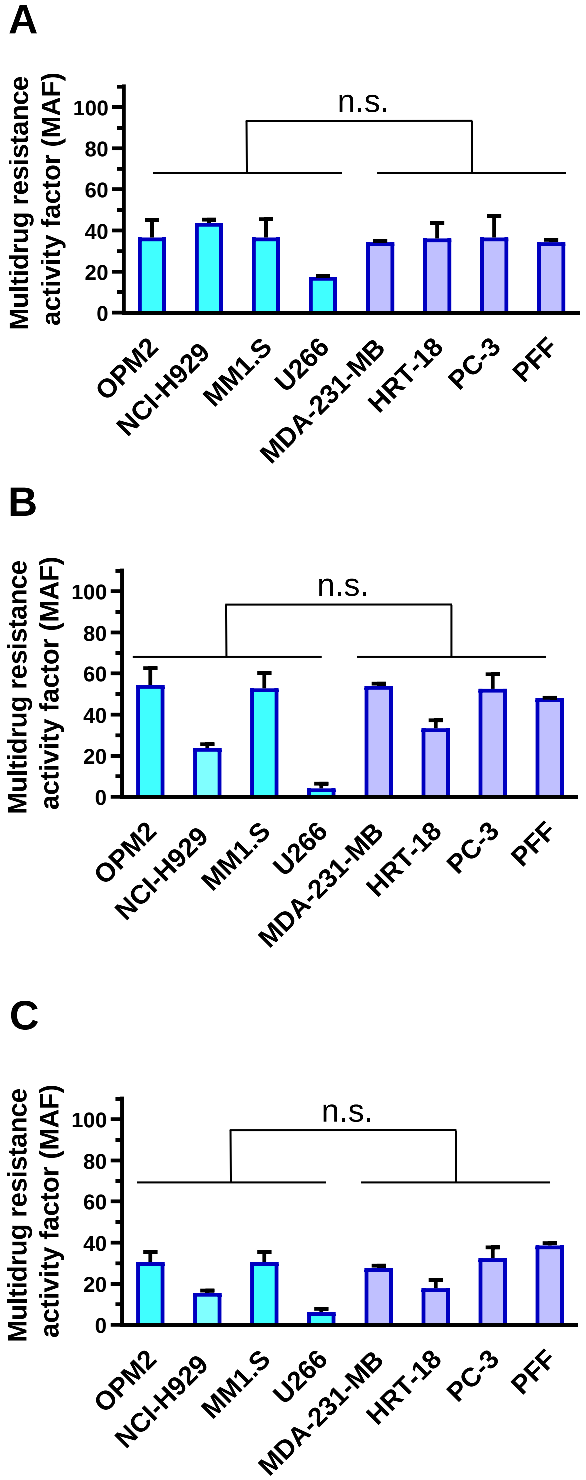

Activity of multidrug resistance proteins in multiple myeloma and solid tumor cell lines

MM, solid tumor cell lines and fibroblasts at equal cell numbers were exposed for 30 min to hydrophobic dye in the presence or absence of transporter specific inhibitors. Cells were treated with (A) verapamil 20µM (specific P-gp inhibitor), (B) 0.05mM MK-571 (specific MRP1/2 inhibitor) and (C) 0.05 mM novobiocin (specific BCRP inhibitor). Thereafter, profiling of three ABC transporters activity was assessed by flow cytometry. Mean of three replicates ± SD is shown.

There was no significant correlation with efflux activity of and sensitivity to BTZ. Only U266 cells displayed lower efflux of hydrophobic drugs.

Figure S5

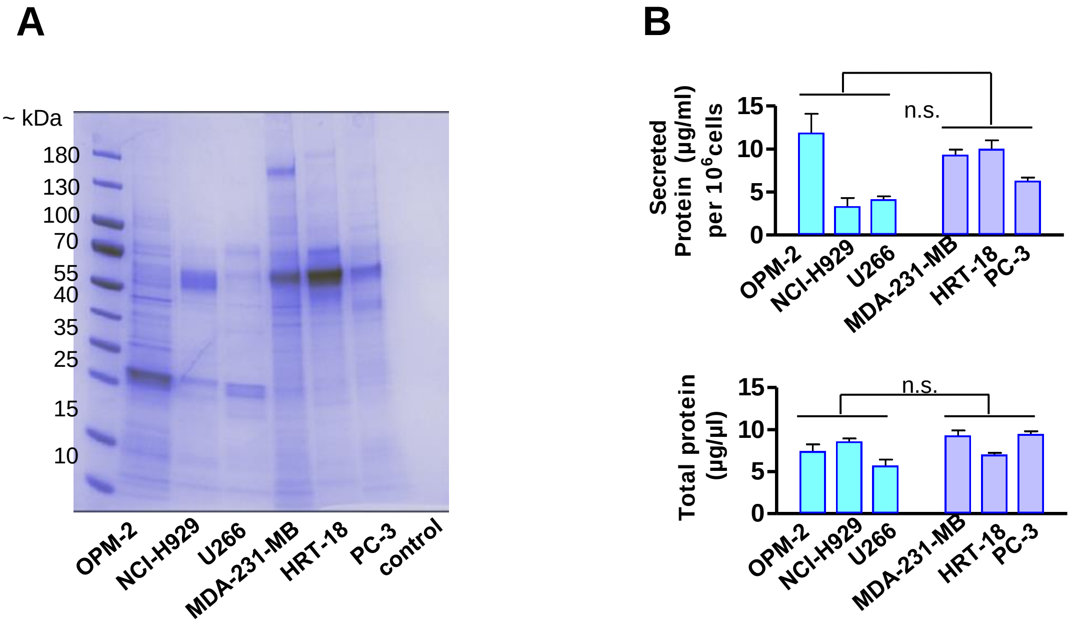

Protein synthesis and secretion by immunoglobulin-producing MM cell lines and BTZ-resistant solid tumor cell lines

(A) SDS-PAGE and subsequent PAGE Blue staining of proteins secreted by cancer cells were done on conditioned protein-free hybridoma medium (PFHM) after 48 hours using an equal number of cells ($2*10^6$) in equal volume (2 ml). Last lane (PFHM) shows negative control, culture medium only. Note: prominent immunoglobulin light chain bands in MM cell lines at 25 kDa

(B) Bradford assay determining the concentration of secreted and total proteins in BTZ-sensitive MM and resistant solid tumor cell lines using equal cell number and volume and subtracting the empty medium background.

BTZ-sensitive MM cell lines have no higher protein synthesis or secretion than BTZ-resistant solid tumor cell lines.

Figure S6

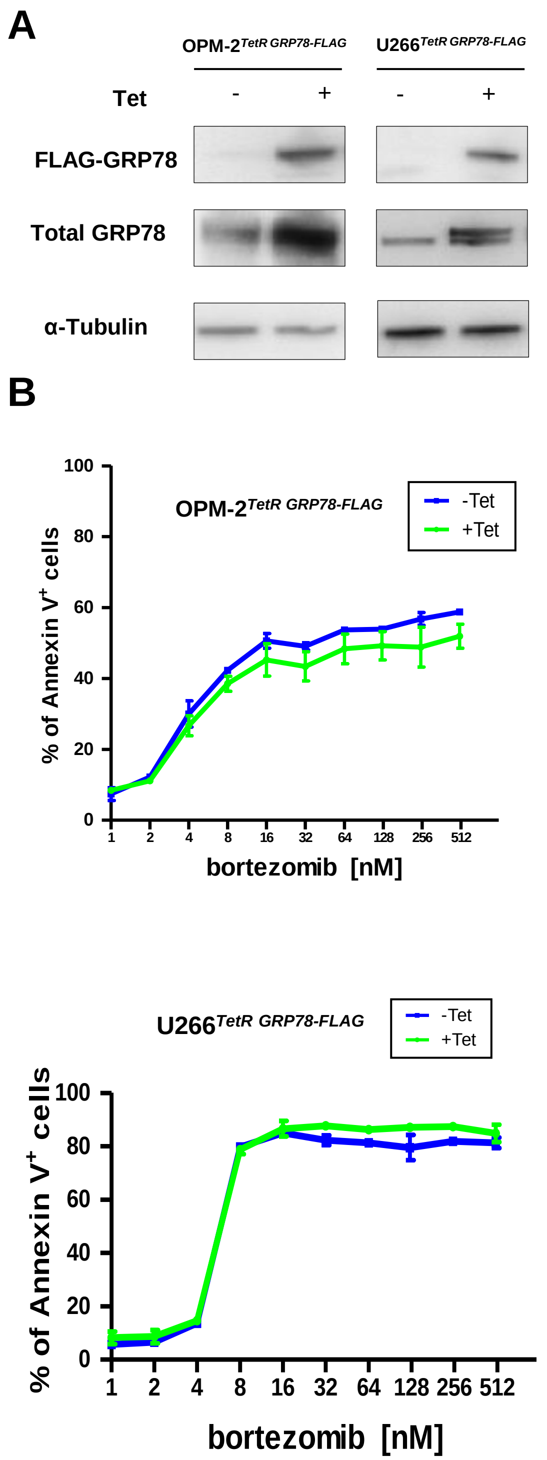

Analysis of GRP78 overexpression and BTZ–induced apoptosis

(A) OPM-2$^{TetR\ GRP78\text{-}FLAG}$ and U266$^{TetR\ GRP78\text{-}FLAG}$ were stimulated with 10 µg/mL tetracycline for 48 h to achieve a robust expression of GRP78-FLAG as monitored by a FLAG-specific antibody. Total GRP78 levels were significantly elevated in OPM-2$^{TetR\ GRP78\text{-}FLAG}$ and U266$^{TetR\ GRP78\text{-}FLAG}$ cells. (B) After induction of GRP78 with 10 µg/mL tetracycline for 48 hours, OPM-2$^{TetR\ GRP78\text{-}FLAG}$ and U266$^{TetR\ GRP78\text{-}FLAG}$ cells were treated with increasing concentrations of BTZ for 24 hours, and cell death was determined by staining with AnnexinV-Per CP eFluor 710 in subsequent flow cytometric analysis.

Overexpression of GRP78 did not protect MM cells from BTZ-induced apoptosis.

Figure S7

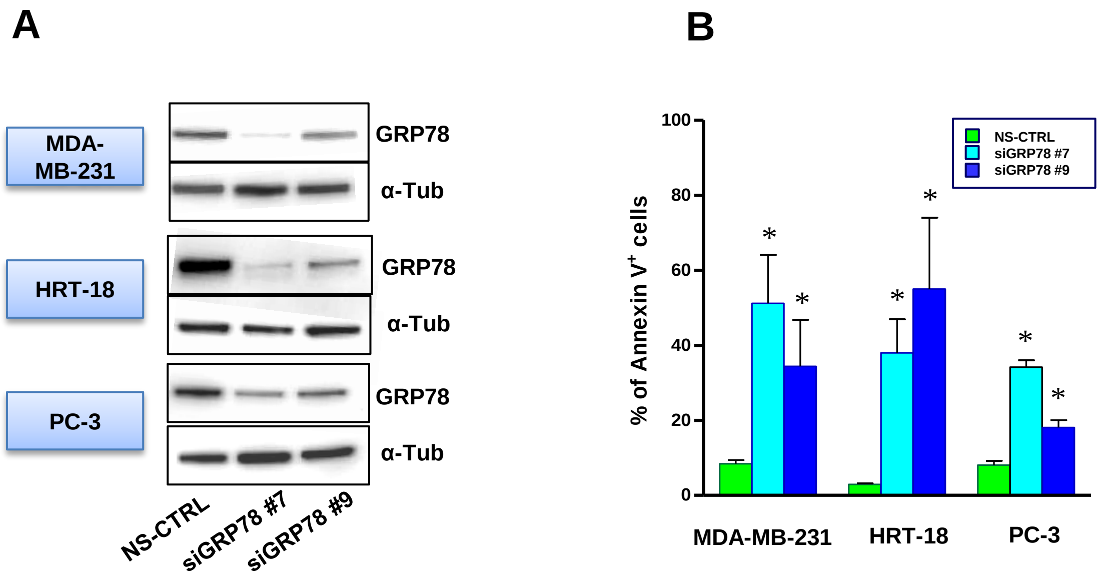

Analysis of GRP78 knockdown and apoptosis of solid tumor cell lines

(A) Solid tumor cell lines (MDA-MB 231, HRT-18 and PC-3) were transiently transfected with two different siRNA sequences targeting the open reading frame of GRP78-specific (siGRP78 #7 and siGRP78 # 9) and non-specific control (NS-CTRL) siRNAs for 72 h. Western blot analysis confirmed a strong knockdown of GRP78 at the protein level with both oligonucleotides. Tubulin alpha served as a control for equal protein loading and transfer.

(B) Following transient siRNA knockdown of GRP78 in MDA-MB-231, HRT-18 and PC-3 cells for 5 days, cell death was analyzed by staining with AnnexinV-Per CP eFluor 710 in subsequent flow cytometry. * indicates $p<0.05$

Knockdown of GRP78 induced apoptosis in solid tumor cell lines.

Figure S8

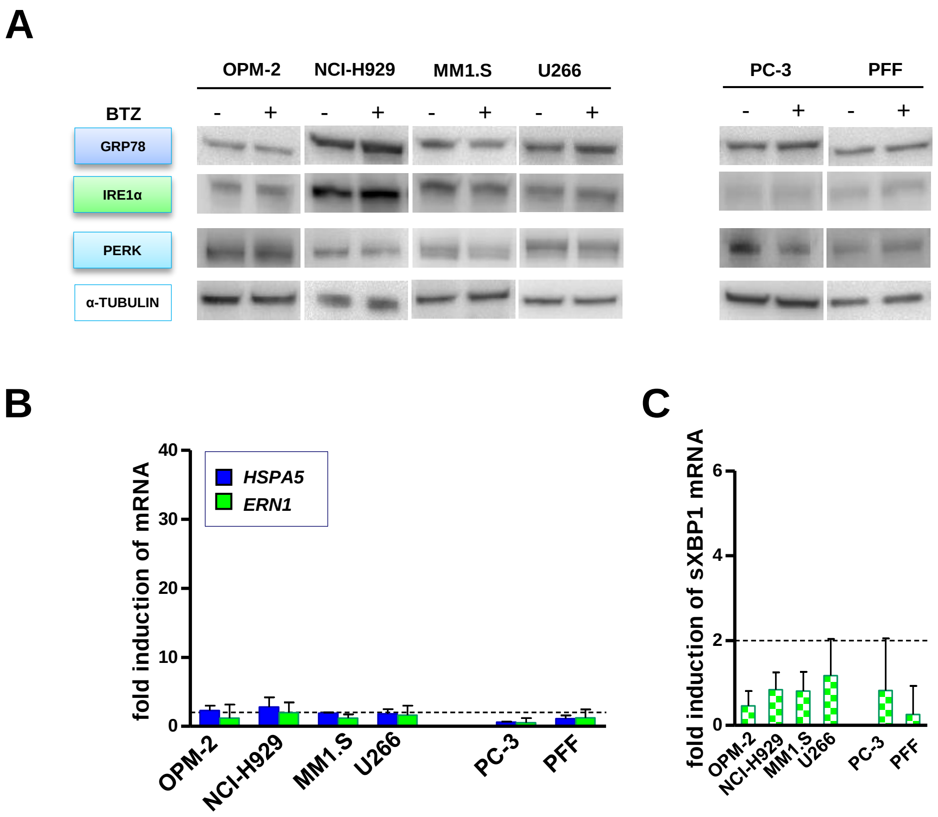

Analysis of canonical UPR after BTZ treatment in MM and solid tumor cells

MM, PC-3 and PFF cells were treated with 10 nM BTZ, and activation of UPR was determined by Western blot analysis (A) and real-time PCR (B/C). (A) Proteins specific to the three main branches of UPR (i.e. upregulation of GRP78 and IRE1α, and PERK phosphorylation) were detected by Western Blot. The data represents at least three repeated experiments.

(B) Moreover, the analysis of gene expression compared to untreated control was done by real-time PCR to monitor *HSPA5* (GRP78), *ERN*1 (IRE1α) and (C) sXBP1 transcription after proteasome inhibition by 10 nM BTZ. Mean ± SD of three independent experiments. * indicates $p<0.05$

BTZ did not induce UPR in MM cell lines, solid tumors cell lines and primary foreskin fibroblasts (PFF).
