# Supplementary material for: Spliced XBP1 Levels Determine Sensitivity of Multiple Myeloma Cells to Proteasome Inhibitor Bortezomib Independent of the Unfolded Protein Response Mediator GRP78
Source: Front Oncol. 2020 Jan 22;9:1530. doi: 10.3389/fonc.2019.01530 (PMC6987373; doi:10.3389/fonc.2019.01530)
Supplement: Supplementary file 3 [file Presentation_2.pdf]

An intact p53/Noxa response and high sXBP1 predominate over endoplasmic reticulum stress in multiple myeloma sensitivity to proteasome inhibition

Bojana Borjan[1,2], Johann Kern[2], Normann Steiner[3], Eberhard Gunsilius[3] and Gerold Untergasser[1,2]

[1]Experimental Oncogenomics Laboratory, Department of Internal Medicine V, Innsbruck Medical University, Innsbruck, Austria; [2]Tyrolean Cancer Research Institute, Innsbruck, Austria; and [3]Department of Internal Medicine V, Innsbruck Medical University, Innsbruck, Austria

Supplementary materials and methods

Substances

Bortezomib was purchased from Selleckchem and dissolved in DMSO (SIGMA Biochemicals) to a stock solution of 50 mM. Thapsigargin was obtained from Molecular Probes and dissolved in DMSO to a stock solution of 1mM.

Patients

According to the International Myeloma Working Group (IMWG) criteria patients with newly diagnosed MM (NDMM) and relapsed/refractory MM (RRMM) were included in the study population (Table 1). Investigations have been conducted in accordance with the ethical standards and according to the Declaration of Helsinki and according to national and international guidelines and have been approved by the authors' institutional review board (AN2015-0034 346/4.13; AN5064 Innsbruck). Bone marrow aspirates (BMA) were subjected to Ficoll-Paque density centrifugation to obtained mononuclear cells. Multiple myeloma plasma cells were purified *ex vivo* from isolated bone marrow mononuclear cells, by magnetic cell sorting (Miltenyl Biotec) according to manufacturer's protocol with anti-human CD138 microbeads. The presence of deletion 17p was assessed by interphase fluorescent in situ hybridization (FISH) and spectral karyotyping (SKY) in all MM samples. Isolated myeloma cells were used for subsequent DNA or RNA extraction. Peripheral blood of healthy donors was centrifuged using Ficoll-Paque, and subjected to the same following steps as bone

marrow samples. Mononuclear cells (MNCs) were isolated and B-cells were enriched by magnetic cell sorting by anti-human CD19 microbeads (Miltenyl Biotec).

Cell culture

The multiple myeloma cell lines OPM-2 (ACC-50; CVCL_1625), NCI-H929 (ACC-136; CVCL_1600) and U266 (ACC-9; CVCL_0566) were purchased from DSMZ Germany, and MM1.S (CRL-2974; CVCL_8792) from ATCC. All used MM cell lines were authenticated by us by flow cytometry (CD138/CD38) and STR-profiling (GeneAnalyzer, ABI). Solid tumor cell lines, prostate cancer PC-3 (CVCL_0035) and breast cancer MDA-MB-231 (CVCL_0062) were obtained from ATTC and colon cancer cell line HRT-18 (CVCL_2514) was purchased from CLS and all authenticated by STR-profiling. Primary

Foreskin Fibroblasts (PFF) were purchased from Promo Cell. Cells were grown in the recommended cell media (RPMI 1640; Lonza) supplemented with 10% fetal calf serum, 105 IU/L penicillin, 100 mg/L streptomycin, and 2 mmol/L glutamine. For analysis of secreted proteins cells were cultivated in Protein-Free hybridoma medium (PFHM-II, Thermo Fisher Scientific) for 48 h.

DNA extraction and next generation sequencing

Genomic DNA was extracted from CD138 enriched cells and used cell lines with the Magnapur nucleic acid isolation station and the nucleic acid extraction kit (provided by the manufacturer (Roche). Concentration of isolated DNA was determined by Qubit 3.0 Fluorometer (Thermo Fisher Scientific). 30 ng of genomic DNA were used to generate libraries for NGS analysis. Quality and quantity of all NGS libraries was assessed by the Bioanalyzer 2010 System and the high sensitivity DNA kit (both Agilent Technologies) prior to multiplexing and cluster generation. Paired-end sequencing was performed with the Miseq Reagent Kit V2 on the Miseq NGS machine (illumina). Fastq files were processed and variants called by the use of TruSeq Amplicon program (BaseSpace Workflow; SCR_011881), 2.0.0.0 Isis (Analysis Software), 2.6.21.7 SAMtools 1.2 (SCR_002105), Isis Smith-Waterman-Gotoh (Aligner), 2.6.21.7 Somatic Variant Caller 4.0.13.1 and IONA (Annotation Service) 1.0.10.37.

Proteasome activity assay

To determine the ß5 subunit proteasome activity in live cells, Proteasome-Glo™ Chymotrypsin-Like, cell-based assays (Promega) was used according to manufacturer's instructions. In brief, equal number of cells (10,000) was plated and incubated with 10nM BTZ for 6h. Reagent containing luminescent substrate specific for chymotrypsin-like site, Suc-LLVY-Glo™, was added to living cells, and after 10 minutes luminescence was recorded with Infinite 200 luminometer (Tecan).

Drug efflux assay

Functional detection and profiling of the activity of three major ABC transporters (p-glycoprotein, MRP1/2 and BCRP) was performed by eFluxx-ID Green multidrug resistance assay kit, Enzo Life Sciences, USA, #ENZ-5109-K100) according to manufacturer's instructions. Cells were incubated with hydrophobic dye for 30 min at 37°C in the presence or absence of specific inhibitors of ABC transporters. Transporter inhibitors used for treatment were verapamil 20μM (specific P-gp inhibitor), 0.05mM MK-571 (specific MRP inhibitor) and 0.05 mM novobiocin (specific BCRP inhibitor). The dye penetrates the cell membrane and undergoes hydrolyses by intracellular esterases. Fluorescent signal was detected by flow cytometry analysis. Untreated cells exhibiting higher activity of multidrug resistant proteins and subsequent increased drug efflux have diminished fluorescence. Inhibitors of transporter proteins induce retention of dye within cells relative to untreated cells. Multidrug resistance activity factor (MAF) was calculated as suggested in kit protocol. The difference in mean fluorescence intensity (MFI) between cells treated and not treated with specific transporter inhibitors is an indication of the corresponding protein activity.

Quantitative RT-PCR analysis

Total RNA was isolated from cell lines and primary bone marrow $CD138^+$ myeloma cells using TRI Reagent (Sigma Biochemicals) according to the manufacturer's instructions. Thereafter, genomic DNA in the RNA samples was digested with the DNAse I (New England Biolabs). The cDNA was amplified from 1 μg total RNA using the SuperScript II Reverse Transcriptase Kit (Invitrogen Life Technologies). For validation, quantitative real time polymerase chain reaction (RT-PCR) was performed

using a SensiMix SYBR No-ROX Kit (Bioline), a Rotor-Gene 6000 detection system (Corbett Research; SCR_017552) and sets of gene-specific primers:
Sets of gene-specific primers were used: 18S 5'-GTTGGTGGAGCGATTTGTCT 5'-GGCCTCACTAAACCATCCAA, *ACTB* (β-Actin) 5'-ATGCAGAAGGAGATCACTGC 5'-CTGCGCAAGTTAGGTTTTGT, sXBP1 5'-AGTCCGCAGCAGGTGCAGGC 5'-GAACTGGGTCCTTCTGGGTAG, *HSPA5* (GRP78) 5'-CTCGACTCGAATTCCAAAGA 5'-AAGGGGACATACATCAAGCA, *BBC3* (PUMA) 5'-TACGAGCGGCGGAGACA 5'-ATGGTGCAGAGAAAGTCCCC, *PMAIP1* (Noxa) 5'-GGACTGTTCGTGTTCAGCTC 5'-CGGAAGTTCAGTTTGTCTCC and *ERN1* (IRE1α) 5'-GGGCGAACAGAATACACCAT 5'- CCATTGGACACAAAGTGGGA.

Western / Dot Blot analysis

Cells were harvested and lysed in a RIPA buffer (Cell Signaling) containing protease inhibitors (Complete Mini EDTA-free; Roche Applied Science). Phosphatase inhibitor cocktail 2 and 3 (Sigma-Aldrich) were added freshly prior to the lysis. Total protein (40 µg) was denatured, separated with 4 -20 % SDS-PAGE (Criterion TGX, Bio-Rad) and transferred to an Immuno-Blot™ polyvinylidene difluoride (PVDF) membrane (Bio-Rad). Membranes were incubated overnight at 4 °C with primary antibodies: mouse anti-α-tubulin (B5-1-2; Sigma Biochemicals; AB_477582), mouse anti-GAPDH (6C5, Santa Cruz; AB_627679), mouse-anti GRP78 (474421, R&D Systems; AB_2233235), rabbit anti-PSMB3 20S proteasome ß3 (PA5-28999, ThermoFisher; AB_2546475), rabbit anti-20S proteasome ß5i (ST1057, Calbiochem; AB_437912), mouse anti-p53 (PAb1801, Calbiochem; AB_10571871) and mouse anti-Noxa (114C307, Calbichem; AB_784877). Rabbit anti-IRE1α (14C10; AB_2055392) and rabbit anti-PERK (C33E10; AB_2095847) were purchased from Cell Signaling. Afterwards, membranes were incubated with an HRP-conjugated secondary antibody (Dako Cytomation) diluted 1:2.500. After washing, a chemoluminescent substrate (LumiGLO Reagent and Peroxide, Cell Signaling Technology) was added to the membrane, which was then exposed in the Chemidoc XRS station (Bio-Rad Laboratories). For ubiquitin Dot Blot assay samples were spotted onto the nitrocellulose membranes (Protean BA 85, GE Healthcare Life Sciences, Whatman) and probed with rabbit anti-ubiquitin, Lys-48 specific antibody (05-1307, Millipore). Myeloma and solid tumor cell lines were incubated in equal cell numbers ($2*10^6$) and equal volumes (2 ml) of protein-free hybridoma medium (PFHM). Conditioned

supernatants with secreted proteins were further concentrated using AmiconUltra-0.5 centrifugal filter unit with ultracel-10 membrane (UFC501096, Milipore). All samples were normalized to cell number and the same volume. Bradford assay was used to determine protein concentrations. Secreted proteins were analyzed by SDS-PAGE with Page Blue Protein staining (Thermo Fisher Scientific).

Flow cytometry

Cell death was evaluated using human PerCP-eFluor® 710-labeled Annexin V (eBiosciences; AB_2575168) staining. Therefore, cells were resuspended in 200 µL Annexin V Binding Buffer (BD Biosciences) with 2 µL Annexin V, incubated for 15 min, washed and resuspended in PBS/ 5% FCS prior to analysis. Cells were examined in the FACSCalibur (Becton-Dickinson, Heidelberg, Germany). Apoptosis was determined by percentage of Annexin V positive cells. Further, all MM cells were verified by surface marker staining with anti-human CD138/CD38 FITC-labeled antibodies (Miltenyl Biotec; AB_2751925/AB_2733810).

Generation of tetracycline-inducible lentiviral GRP78-FLAG overexpression system in myeloma cells

Myeloma cell lines (OPM-2TetR) were generated as described elsewhere (18). In brief, OPM-2 cells were cultivated in RPMI1640 containing 10% tetracycline-free FCS (Clontech). Parental line OPM-2 was lentivirally transfected (pLENTI6/TR, Thermo Fisher Scientific) and selected with 1 mg/mL neomycin (Biochrom) to express the tetracycline repressor (TR) protein. pLenti 6.3/ GRP78-FLAG lentiviruses were generated after cloning the HSPA5 cDNA (BC020235, GE-Dharmacon) into the p3x FLAG CMV 14 expression vector (SIGMA Biochemicals) by the use of primers, amplifying the open reading frame (GRP78-for: 5-gatatcgcaggctccaccatg; GRP78-rev: 5-ggatccttgcagataattgg), and the KOD polymerase (Calbiochem). The GRP78-FLAG coding sequence was cut out from the p3x FLAG CMV14 vector and ligated into the Gateway pENTR 11 Dual Selection vector – Thermo Fisher Scientific. An expression clone was generated by performing a recombination reaction between the entry vector pENTR 11/GRP78-FLAG and a Gateway destination vector (pLenti6.3/V5–DEST) with the use of the GatewayR LR Clonase™ II Enzyme mix (Thermo Fisher Scientific). Lentiviral particles were produced by transient transfection of HEK293FT cells with replication defective lentivirus recombinant DNA

(pLenti6.3/GRP78-FLAG) along with Viral Power mix (all Thermo Fisher Scientific) using Lipofectamine 2000 (Invitrogen). Selected OPM-2TetR line was further lentivirally transfected (pLenti6.3/ GRP78-FLAG) and stable cell line was selected with 2.5 µg/ml blasticidin (Invitrogen). OPM-2$^{TetR\ GRP78\text{-}FLAG}$ cells were propagated in 10% tetracycline-free FCS (Clontech).

Immunofluorescence and confocal microscopy

Myeloma cell line (OPM-2$^{TetR\ GRP78\text{-}FLAG}$) was exposed to 10 µg/ml tetracycline and allowed to express GRP78-FLAG for 48 h. Myeloma cell suspension was centrifuged onto the glass slides using Cytospin3 (Shanondon) cytocentrifuge machine. Air-dried preparations were fixed in cold methanol: acetone − 1:1 (v/v), permeabilized with 0.2% Triton X-100 in PBS and incubated overnight in a humidified chamber at 4 °C with primary mouse anti-FLAG antibody (F1804, Sigma; AB_262044) at the 1:1000 dilution in 1% BSA. After rinsing in PBS, cells were incubated with 1:1000 diluted Alexa Fluor 488-–conjugated goat anti-rabbit secondary antibody (A11029, Life Technologies) and cell nuclei were counterstained with DAPI. Imaging was conducted with spinning disc confocal microscopic system with Velocity software (Ultra VIEW VoX; Perkin Elmer) connected to a Zeiss Axio Observer Z1 inverted microscope.

RNA interference

Two independent siRNA sequences siGRP78#7 (sense: 5‘-ggagcgcauugauacuaga antisense: 5’-ucuaguaucaaugcgcucc) and siGRP78#9 (sense: 5’-gccuaaauguuaugaggauca antisense: 5’-ugauccucauaacauuuaggc), targeting different regions of the GRP78 mRNA, were used to knock down (downregulate) gene expression in MDA-MB-231, HRT-18 and PC-3 cells. Cells were transfected either with GRP78 siRNA or non-specific control siRNA at the final concentration of 80 nM using Lipofectamin 2000 reagent (Invitrogen) in serum-reduced culture medium. Transfection procedures with repeated transfections were applied for each line.

Immunoprecipitation of unfolded proteins

To detect unfolded proteins in ER, stable myeloma cell line OPM-2TetR GRP78-FLAG with tetracycline-inducible overexpression was grown in RPMI-1640 medium

containing 10 µg/ml tetracycline for 48 h to express GRP78-FLAG prior to addition of 10 nM BTZ for the time course of 16 h. Cells were harvested in diluted low detergent RIPA Buffer (0.01% NP-40, Cell Signaling) and then subjected to immunoprecipitation using anti-FLAG M2 affinity gel (A2220, Sigma-Aldrich). Purified murine anti-FLAG monoclonal antibody covalently attached to agarose beads was incubated with the cell lysates overnight at 4 °C. The overexpressed GRP78-FLAG and interacting unfolded proteins were eluted under native conditions by an excess of 3xFLAG peptide (Sigma-Aldrich). Thereafter, protein was denatured and misfolded ʎ light chains were analyzed by SDS-PAGE and immunoblotting using rabbit anti-ʎ light chain antibody (ab109247, Abcam; AB_10863272).

Statistical analysis

Statistical analyses were performed with the GraphPad Prism™ (SCR_002798) software for Windows. Student’s T test 2-tailed, two-way ANOVA and Mann-Whitney U Tests were used to study differences between groups.
